# Rectal Cancer Complicated by a Pelvic Arteriovenous Malformation Managed with Preoperative Embolization and Robotic Surgery: A Case Report

**DOI:** 10.70352/scrj.cr.26-0030

**Published:** 2026-04-18

**Authors:** Kazunosuke Yamada, Shinsuke Takeno, Yusuke Araki, Takuya Hara, Eiji Furukoji, Atsushi Nanashima

**Affiliations:** 1 Department of Surgery, University of Miyazaki, Miyazaki, Miyazaki, Japan; 2 Department of Radiology, University of Miyazaki, Miyazaki, Miyazaki, Japan

**Keywords:** rectal cancer, pelvic arteriovenous malformation, preoperative embolization, robotic surgery, indocyanine green fluorescence angiography

## Abstract

**INTRODUCTION:**

Pelvic arteriovenous malformations (AVMs) are rare vascular anomalies that may cause marked venous engorgement and increase the risk of massive hemorrhage during pelvic surgery. When rectal cancer coexists with a pelvic AVM, achieving oncological radicality while maintaining surgical safety becomes particularly challenging.

**CASE PRESENTATION:**

A patient with rectal cancer complicated by a pelvic AVM was referred for surgical treatment. Preoperative imaging demonstrated a pelvic AVM supplied by branches of the internal iliac artery with venous drainage into the rectal venous plexus. Selective preoperative coil embolization was performed to treat the AVM while rectal arterial perfusion was preserved. Robotic-assisted total mesorectal excision (TME) was subsequently undertaken. Despite residual presacral venous dilatation related to chronic hemodynamic changes, robotic-assisted TME was completed without hemorrhagic complications. Intraoperative indocyanine green fluorescence angiography confirmed adequate perfusion of both the proximal colon and the distal rectal stump, allowing safe primary anastomosis without a diverting stoma. The postoperative course was uneventful.

**CONCLUSIONS:**

Rectal cancer associated with a pelvic AVM presents unique surgical challenges due to altered pelvic vascular anatomy. This case suggests that careful preoperative planning, selective embolization, and appropriate integration of advanced surgical techniques may facilitate safe radical resection in similarly complex pelvic conditions.

## Abbreviations

AVM
: arteriovenous malformation
CTA
: CT angiography
ICG
: indocyanine green
LAR
: low anterior resection
TME
: total mesorectal excision

## INTRODUCTION

Pelvic AVMs are rare congenital vascular anomalies characterized by high-flow arteriovenous shunts through an abnormal vascular network known as a nidus. Their incidence is extremely low, and clinical manifestations are heterogeneous, ranging from pelvic or perineal pain and urogenital symptoms to, in rare cases, high-output cardiac failure.^1,2)^ From a surgical perspective, pelvic AVMs are particularly problematic because the associated venous engorgement and fragile collateral vessels can substantially increase the risk of intraoperative hemorrhage. Marked dilatation of the pelvic venous plexus may lead to spontaneous rupture or massive bleeding during pelvic surgery, representing a potentially life-threatening complication. With advances in endovascular techniques, selective embolization using N-butyl-2-cyanoacrylate or coils has become the mainstay of treatment for pelvic AVMs, with favorable long-term outcomes reported.^3–7)^ Pelvic AVMs are typically supplied by branches of the internal iliac artery and are located within the pelvic soft tissue, features that distinguish them anatomically and hemodynamically from mesenteric or gastrointestinal AVMs arising from mesenteric arterial branches and involving the bowel wall. This distinction is clinically important because it directly influences the therapeutic strategy, particularly when colorectal resection is planned.^8–10)^

LAR with TME is one of the most technically demanding procedures in colorectal surgery. Injury to the presacral venous plexus during posterior dissection can result in massive bleeding that is difficult to control, and this risk is expected to be further increased in the presence of a pelvic AVM.^11)^ Robotic surgery offers technical advantages in deep pelvic dissection, including high-definition 3D visualization and articulated instruments, which may enhance surgical safety in anatomically complex situations.^11–13)^ Anastomotic leakage remains one of the most serious complications after LAR. Although inadequate perfusion is a major contributing factor, intraoperative assessment based solely on macroscopic appearance is subjective. ICG fluorescence angiography has therefore been introduced as an adjunctive tool for real-time assessment of bowel microperfusion. While randomized trials have shown that ICG fluorescence angiography can influence intraoperative decision-making, its impact on anastomotic leakage rates remains inconsistent.^14–16)^

A literature search was conducted using the PubMed database for articles published up to 2025 using the keywords “rectal cancer” and “pelvic arteriovenous malformation.” To the best of our knowledge, no previous reports have specifically described rectal cancer complicated by a pelvic AVM. A recent case report described sigmoid colon cancer associated with a pelvic AVM treated with preoperative embolization followed by laparoscopic surgery; however, reports describing colorectal cancer surgery in the presence of a pelvic AVM remain extremely limited.^17)^

Herein, we report a rare and surgically challenging case in which rectal cancer complicated by a pelvic AVM was successfully managed using a staged strategy consisting of preoperative embolization, robotic-assisted TME, and intraoperative ICG fluorescence angiography, enabling safe oncologic resection and primary anastomosis.

## CASE PRESENTATION

A 69-year-old woman was referred to our institution for further evaluation of hematochezia detected at a previous outpatient clinic visit. She had no significant past medical history, was not taking any medications, and had no history of smoking or alcohol abuse. Her abdomen was soft and non-tender without palpable masses, and digital rectal examination did not reveal a palpable tumor. Preoperative blood tests showed mild anemia (hemoglobin 11.2 g/dL), whereas liver and renal function, coagulation parameters, and nutritional indices were within normal limits.

**Fig. 1:**
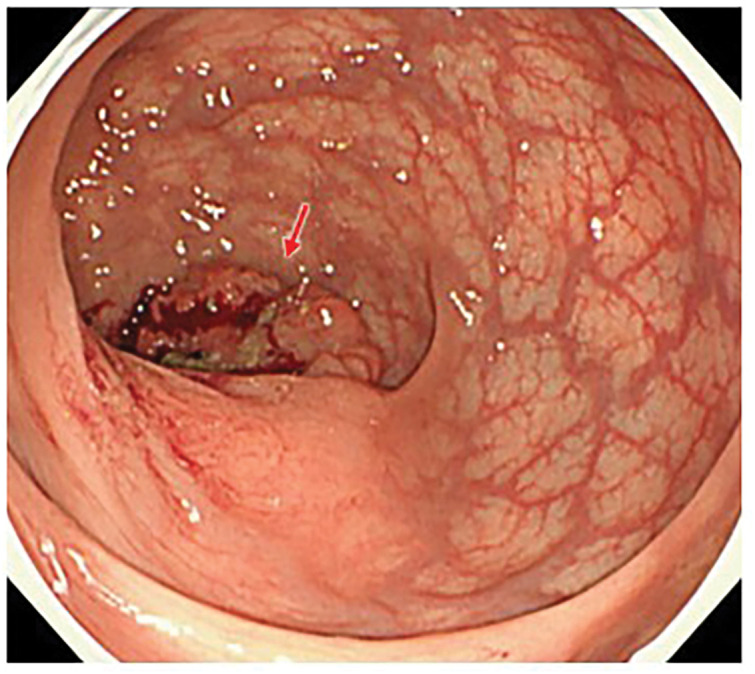
Colonoscopic findings of the rectal tumor. Type 2 tumor in the middle rectum (≈10 cm from the anal verge). The red arrow indicates the lesion.

Colonoscopy revealed a 50-mm type 2 lesion located approximately 10 cm from the anal verge, corresponding to the middle rectum, and involving about one-half to two-thirds of the circumference, without luminal stenosis (**Fig. 1**). Biopsy specimens demonstrated a well to moderately differentiated tubular adenocarcinoma.

**Fig. 2:**
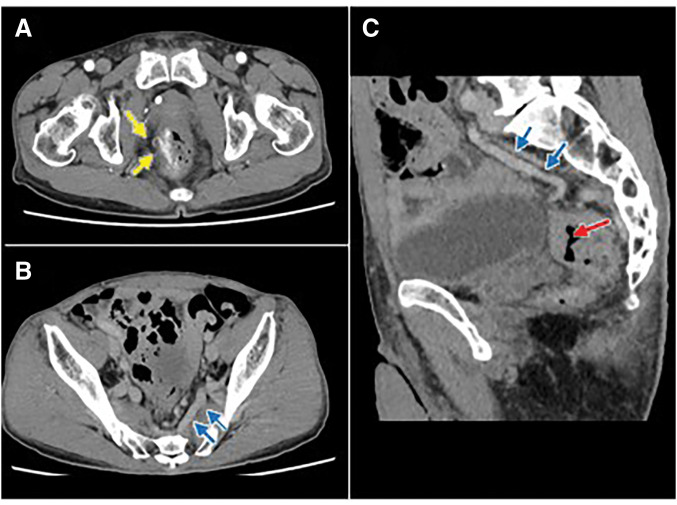
Contrast-enhanced CT demonstrating the rectal tumor and pelvic AVM. (**A**) Axial CT showing the nidus. (**B**) Axial CT showing dilated draining veins. (**C**) Sagittal CT demonstrating the rectal tumor and draining veins. Yellow arrows indicate the nidus; blue arrows indicate draining veins; the red arrow indicates the tumor. AVM, arteriovenous malformation

**Fig. 3:**
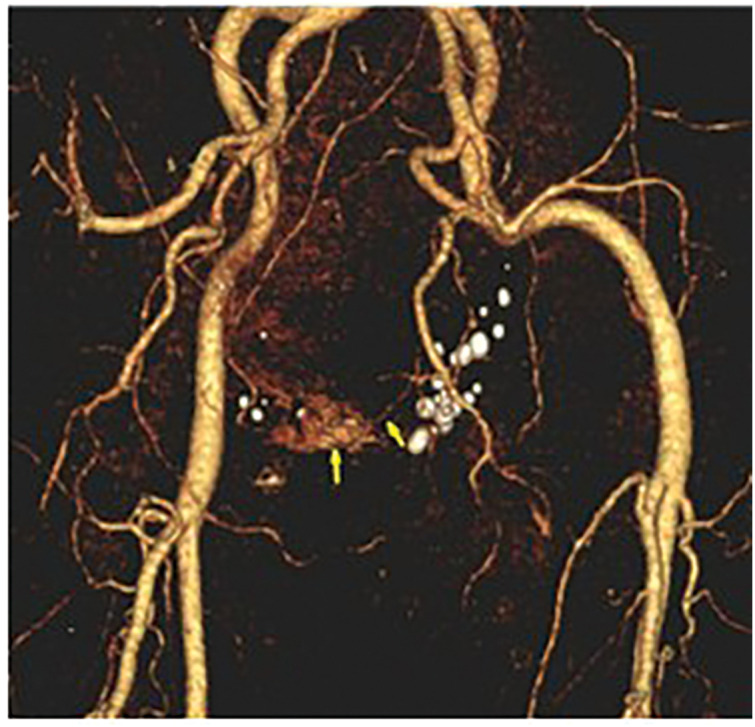
3D-CTA of the pelvic AVM. 3D-CTA demonstrating the nidus of the pelvic AVM (yellow arrows). The right side of the image corresponds to the patient’s right side, viewed from a posterior perspective. AVM, arteriovenous malformation; CTA, CT angiography

Contrast-enhanced CT demonstrated a rectal tumor in the middle rectum with regional lymphadenopathy, and the clinical diagnosis was cT3N1aM0, Stage IIIA. In addition, arterial-phase images revealed a strongly enhancing nidus located on the right side external to the wall of the lower rectum, supplied by a branch of the right internal pudendal artery, consistent with a pelvic AVM (**Fig. 2**). The draining veins extended from the rectal venous plexus into the bilateral internal iliac veins and the inferior mesenteric vein and were dilated, accompanied by multiple phleboliths, indicating a high-flow arteriovenous shunt. 3D-CTA clearly depicted the spatial relationship among the nidus, feeding arteries, and draining veins, confirming a pelvic AVM predominantly supplied by the right internal pudendal artery (**Fig. 3**).

**Fig. 4:**
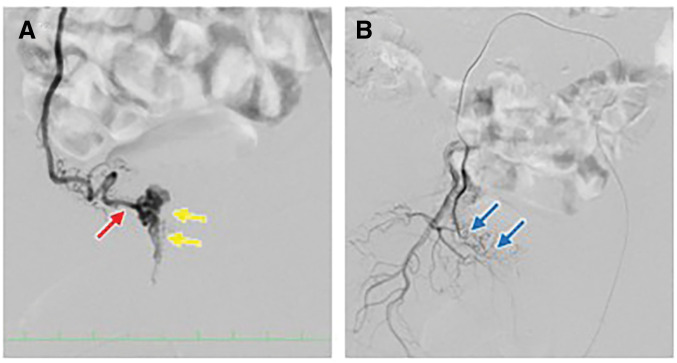
Selective angiography before and after coil embolization of the pelvic AVM. (**A**) Pre-embolization angiogram showing the nidus of the pelvic AVM (yellow arrows) and the feeding artery (red arrow). (**B**) Post-embolization angiogram showing complete occlusion of the nidus, with coils visible as high-density signals (blue arrows). AVM, arteriovenous malformation

To reduce the risk of intraoperative hemorrhage and to facilitate pelvic dissection, selective preoperative embolization was planned. Three days before the scheduled rectal resection, selective transarterial embolization was performed. Angiography of the right internal iliac artery demonstrated a type 1 AVM according to the Cho classification, mainly supplied by the right internal pudendal artery. Coil embolization was performed using detachable coils (i-ED Coil; Kaneka, Osaka, Japan), with a total of 10 coils deployed to selectively occlude the feeding branches. After embolization, the nidus and dilated draining veins were no longer visualized, indicating effective elimination of the arteriovenous shunt, and no procedure-related complications occurred (**Fig. 4**). No additional embolization or further angiographic evaluation of the contralateral internal iliac artery or other potential collateral feeders was performed, as the embolization effect was considered sufficient based on angiographic findings.

Robotic-assisted LAR was subsequently performed using the da Vinci Xi system (Intuitive Surgical, Sunnyvale, CA, USA). A standard medial-to-lateral approach was employed, with ligation of the inferior mesenteric artery at its origin followed by division of the inferior mesenteric vein. The inferior mesenteric vein, which had been dilated on preoperative imaging, had returned to a near-normal caliber at the time of surgery after embolization.

**Fig. 5:**
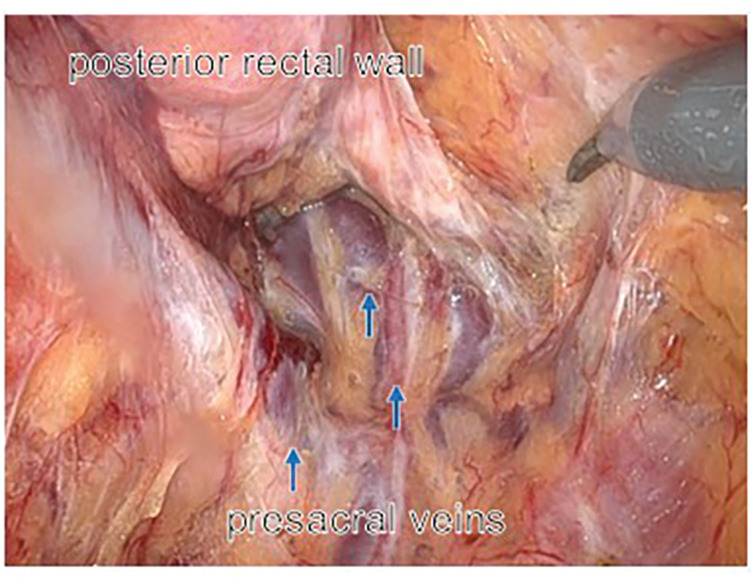
Intraoperative view of the posterior rectal wall and presacral veins. Robotic dissection of the posterior plane during TME. The posterior rectal wall is labeled, and mildly dilated presacral veins are indicated by blue arrows. TME, total mesorectal excision

During pelvic dissection, dilated presacral veins were still observed. Although the dilatation of the inferior mesenteric vein showed improvement after embolization, it was not possible to directly assess changes in the presacral venous dilatation intraoperatively, as these veins were considered to reflect long-standing structural changes secondary to chronic high-flow hemodynamics (**Fig. 5**). Nevertheless, TME was completed along the correct anatomical plane without uncontrollable bleeding.

Intraoperative digital rectal examination revealed a firm area corresponding to the embolization coil mass in the lower rectum. Intraoperative colonoscopy confirmed that the rectal tumor was located sufficiently proximal to the embolized segment, allowing a safe distal resection margin to be secured.

**Fig. 6:**
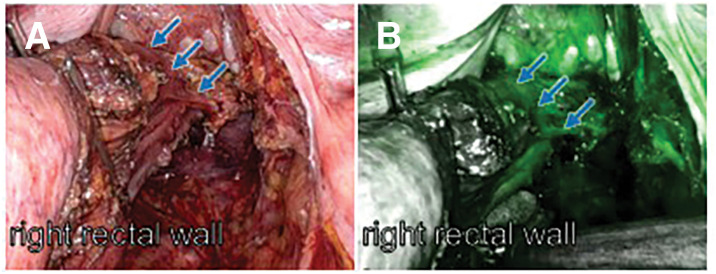
Intraoperative assessment of perfusion of the planned rectal transection line using ICG fluorescence. (**A**) White-light view after mesorectal division showing the right rectal wall (blue arrows). (**B**) ICG fluorescence imaging confirming adequate perfusion along the planned rectal transection line on the right rectal wall (blue arrows). ICG, indocyanine green

After completion of mesenteric division and before final rectal transection, intraoperative ICG fluorescence angiography was performed to assess bowel perfusion along the planned transection line. ICG (5 mg) was administered intravenously, and homogeneous, circumferential fluorescence was observed at the planned transection line as well as along the preserved distal rectal stump, with no areas of delayed or absent perfusion (**Fig. 6**). Based on these findings, the planned transection line was considered appropriate.

Rectal transection was subsequently performed at the mid-rectum level, approximately 4 cm distal to the peritoneal reflection. Colorectal anastomosis was then created using the double-stapling technique with a 25-mm circular stapler (CDH 25; Ethicon, Somerville, NJ, USA) without a diverting stoma.

Histopathological examination demonstrated moderately differentiated tubular adenocarcinoma (pT3N0, pStage IIa), with no lymphatic or perineural invasion. The circumferential resection margin was negative, and an R0 resection was achieved. No AVM-related vascular abnormalities were identified within the bowel wall or mesentery.

The postoperative course was uneventful. Oral intake was resumed gradually, and no postoperative bleeding, anastomotic leakage, or other complications occurred. The patient was discharged on POD 17. At 1-year follow-up, imaging studies demonstrated no evidence of tumor recurrence or residual or recurrent pelvic AVM.

## DISCUSSION

Rectal cancer associated with a pelvic AVM is rare and technically challenging because abnormal pelvic vasculature increases the risk of bleeding during TME. In the present case, the feeding artery from the internal pudendal artery and venous drainage into the rectal venous plexus supported the diagnosis of a pelvic AVM rather than a bowel wall lesion, which was further confirmed by the absence of intramural vascular abnormalities on histopathological examination.^12)^ This distinction was clinically important because pelvic AVMs are amenable to targeted endovascular embolization, whereas mesenteric or intramural AVMs often require intestinal resection.^8–10)^

The primary objective of preoperative embolization in this case was effective management of the pelvic AVM, while simultaneously minimizing the impact on rectal arterial perfusion in anticipation of subsequent rectal resection. Because the feeding vessels of the AVM were not completely independent of the arterial supply to the rectum, an embolization strategy prioritizing both eradication of the nidus and preservation of rectal blood flow was required. Although liquid embolic agents such as N-butyl-2-cyanoacrylate are effective for extensive nidus penetration, they may affect adjacent microvascular territories and carry a risk of non-target embolization. In contrast, selective coil embolization allows precise occlusion of the feeding arteries while preserving the surrounding vascular supply, including potential contributors to rectal perfusion.^18,19)^ This approach resulted in effective AVM control while maintaining sufficient perfusion for safe colorectal anastomosis.

Despite successful eradication of the pelvic AVM by preoperative embolization, dilated presacral veins resulting from long-standing high-flow hemodynamics remained evident intraoperatively. This finding indicated that, although the pathological shunt had been eliminated, structural venous changes persisted and continued to pose a potential risk of venous injury during pelvic dissection.^20)^ In such anatomically complex situations, the robotic platform may provide technical advantages during deep pelvic dissection. In the present case, dilated veins were located along the curved surface of the sacrum, and dissection in this region required delicate manipulation while avoiding traction on fragile venous structures. The articulated instruments of the robotic system allow a more natural approach angle along the presacral curvature. These characteristics may facilitate precise dissection along the mesorectal plane while minimizing traction on fragile venous structures. Although similar procedures may be achievable using conventional laparoscopic techniques, robotic surgery may serve as a useful facilitating tool in anatomically complex pelvic environments by providing enhanced dexterity, tremor filtration, and stable 3D visualization.^11–13)^ In the present case, the safe completion of TME without hemorrhagic complications likely reflects the combined effects of preoperative embolization and careful surgical technique.

Another potential consideration in the management of rectal cancer is neoadjuvant chemoradiotherapy or total neoadjuvant therapy, which is widely used for locally advanced rectal cancer. In the present case, however, the tumor was located in the mid-rectum (approximately 10 cm from the anal verge), close to the peritoneal reflection, and preoperative imaging did not suggest circumferential resection margin involvement. In addition, the presence of a pelvic AVM posed a potential technical challenge for pelvic surgery. If radiotherapy had been administered and a complete clinical response had not been achieved, subsequent surgery might have become substantially more difficult because of radiation-induced fibrosis in an already anatomically complex pelvis. Therefore, upfront surgical resection following selective embolization was considered the most appropriate treatment strategy in this case.

A further theoretical consideration is the potential influence of chronic venous hypertension associated with pelvic AVMs on lymphatic drainage. Persistent elevation of venous pressure may alter interstitial fluid dynamics and could theoretically influence lymphatic flow patterns. The lymphatic system plays a fundamental role in maintaining tissue fluid homeostasis and returning interstitial fluid to the systemic circulation, suggesting that alterations in local hemodynamics may affect lymphatic transport mechanisms.^21)^ However, direct evidence regarding the influence of pelvic AVMs on lymphatic drainage or patterns of nodal spread in rectal cancer remains limited. In the present case, the tumor was located in the mid-rectum (approximately 10 cm from the anal verge), close to the peritoneal reflection. According to current surgical practice in Japan, lateral pelvic lymph node dissection is not routinely indicated for tumors at this level in the absence of radiologically suspicious nodes. Preoperative CT in this patient demonstrated no enlarged lymph nodes in the lateral pelvic compartment. Standard TME was therefore performed according to established oncological principles, and pathological examination revealed no lymph node metastasis. Furthermore, histopathological evaluation of the resected specimen did not demonstrate lymphatic invasion or structural abnormalities of lymphatic vessels. However, because pathological assessment was limited to the mesorectal specimen obtained by TME, evaluation was restricted to the superior lymphatic drainage pathway. Potential alterations in the lateral pelvic lymphatic pathways could not be assessed in the present case. Therefore, although no abnormalities were identified in the examined specimen, the possible influence of AVM-related hemodynamic changes on lateral pelvic lymphatic drainage cannot be completely excluded. Accumulation of additional cases may help clarify whether chronic vascular abnormalities influence lymphatic flow patterns or the distribution of lymph node metastasis in rectal cancer.

Anastomotic leakage remains one of the most serious complications after LAR and is associated with adverse short- and long-term outcomes, including increased local recurrence rates.^22)^ Although impaired perfusion is widely recognized as a major contributing factor, intraoperative assessment based solely on macroscopic appearance is inherently subjective. ICG fluorescence angiography has therefore been introduced as an adjunctive tool for real-time evaluation of bowel perfusion. However, most previous randomized controlled trials have primarily focused on assessment of the proximal bowel segment, and the perfusion status of the distal rectal stump has been less extensively evaluated.^14–16)^ In the present case, preoperative embolization altered pelvic vascular dynamics, making careful intraoperative assessment of bowel perfusion particularly important. Intraoperative ICG fluorescence angiography confirmed adequate perfusion at the planned transection line and the preserved rectal stump. Because a diverting stoma is not always mandatory in mid-rectal cancer surgery, confirmation of adequate perfusion by ICG provided additional reassurance when performing primary anastomosis in this hemodynamically altered pelvic environment. Nevertheless, this observation is based on a single case, and ICG should be interpreted as an adjunctive assessment tool rather than a definitive determinant for surgical decision-making.

## CONCLUSIONS

In this case, a combination of preoperative embolization, robotic-assisted TME, and intraoperative ICG fluorescence angiography enabled safe radical resection with primary anastomosis despite complex pelvic vascular anatomy.

## DECLARATIONS

### Availability of data and materials

The datasets generated and/or analyzed during the current study are available from the corresponding author upon reasonable request.

## References

[1] Annam A. Female pelvic vascular malformations. Semin Intervent Radiol 2018; 35: 62–8. 29628618 10.1055/s-0038-1636524 PMC5886763

[2] Christenson BM, Gipson MG, Smith MT. Pelvic vascular malformations. Semin Intervent Radiol 2013; 30: 364–71. 24436563 10.1055/s-0033-1359730 PMC3835586

[3] Jacobowitz GR, Rosen RJ, Rockman CB, et al. Transcatheter embolization of complex pelvic vascular malformations: results and long-term follow-up. J Vasc Surg 2001; 33: 51–5. 11137923 10.1067/mva.2001.111738

[4] Mallios A, Laurian C, Houbballah R, et al. Curative treatment of pelvic arteriovenous malformation–an alternative strategy: transvenous intra-operative embolisation. Eur J Vasc Endovasc Surg 2011; 41: 548–53. 21277234 10.1016/j.ejvs.2010.11.018

[5] Erbahceci Salik A, Islim F, Akgul A, et al. Concomitant transarterial and transvenous embolization of a pelvic arteriovenous malformation using a new liquid embolic agent, squid-12 and detachable coils. Case Rep Vasc Med 2014; 2014: 972870. 25180118 10.1155/2014/972870 PMC4142281

[6] Zabicki B, Holstad MJV, Limphaibool N, et al. Endovascular therapy of arteriovenous malformation in a male patient with severe post-coital pelvic pain. Pol J Radiol 2019; 84: e258–61. 31481998 10.5114/pjr.2019.86893 PMC6717946

[7] Cleary CM, Masood M, Boutrous ML, et al. Endovascular management of a pelvic arteriovenous malformation. J Vasc Surg Cases Innov Tech 2022; 8: 736–9. 36425253 10.1016/j.jvscit.2022.10.008 PMC9678973

[8] Justaniah AI, Molgaard C, Flacke S, et al. Congenital inferior mesenteric arteriovenous malformation presenting with ischemic colitis: endovascular treatment. J Vasc Interv Radiol 2013; 24: 1761–3. 24160837 10.1016/j.jvir.2013.05.064

[9] Noor M, Cooper K, Lujan H, et al. Arteriovenous malformation of the inferior mesenteric artery presenting as ischemic colitis. Vasc Med 2016; 21: 555–7. 27138349 10.1177/1358863X16645855

[10] Kimura Y, Hara T, Nagao R, et al. Natural history of inferior mesenteric arteriovenous malformation that led to ischemic colitis: a case report. World J Clin Cases 2021; 9: 396–402. 33521107 10.12998/wjcc.v9.i2.396 PMC7812891

[11] Lam J, Tam MS, Retting RL, et al. Robotic versus laparoscopic surgery for rectal cancer: a comprehensive review of oncological outcomes. Perm J 2021; 25: 21.050. 10.7812/TPP/21.050 PMC8782436 35348098

[12] Chaouch MA, Hussain MI, Carneiro da Costa A, et al. Robotic versus laparoscopic total mesorectal excision with lateral lymph node dissection for advanced rectal cancer: a systematic review and meta-analysis. PLoS One 2024; 19: e0304031. 38809911 10.1371/journal.pone.0304031 PMC11135705

[13] Zou J, Zhu H, Tang Y, et al. Robotic versus laparoscopic surgery for rectal cancer: an updated systematic review and meta-analysis of randomized controlled trials. BMC Surg 2025; 25: 86. 40022103 10.1186/s12893-025-02805-z PMC11869447

[14] Alekseev M, Rybakov E, Shelygin Y, et al. A study investigating the perfusion of colorectal anastomoses using fluorescence angiography: results of the FLAG randomized trial. Colorectal Dis 2020; 22: 1147–53. 32189424 10.1111/codi.15037

[15] De Nardi P, Elmore U, Maggi G, et al. Intraoperative angiography with indocyanine green to assess anastomosis perfusion in patients undergoing laparoscopic colorectal resection: results of a multicenter randomized controlled trial. Surg Endosc 2020; 34: 53–60. 30903276 10.1007/s00464-019-06730-0

[16] Jayne D, Croft J, Corrigan N, et al. Intraoperative fluorescence angiography with indocyanine green to prevent anastomotic leak in rectal cancer surgery (IntAct): an unblinded randomised controlled trial. Lancet Gastroenterol Hepatol 2025; 10: 806–17. 40690925 10.1016/S2468-1253(25)00101-3

[17] Inaguma G, Otsuka K, Masumori K, et al. Laparoscopic sigmoidectomy in a male colon cancer patient with pelvic arteriovenous malformation using preoperative interventional radiology: a case report. Asian J Endosc Surg 2025; 18: e70037. 39978930 10.1111/ases.70037 PMC11842171

[18] Kurata A, Suzuki S, Ozawa H, et al. Application of the liquid coil as an embolic material for arteriovenous malformations. Interv Neuroradiol 2005; 11: 287–95. 20584489 10.1177/159101990501100315 PMC3404786

[19] Pal A, Blanzy J, Gómez KJR, et al. Liquid embolic agents for endovascular embolization: a review. Gels 2023; 9: 378. 37232970 10.3390/gels9050378 PMC10217684

[20] Casal Núñez JE, Vigorita V, Ruano Poblador A, et al. Presacral venous bleeding during mobilization in rectal cancer. World J Gastroenterol 2017; 23: 1712–9. 28321171 10.3748/wjg.v23.i9.1712 PMC5340822

[21] Mehrara BJ, Radtke AJ, Randolph GJ, et al. The emerging importance of lymphatics in health and disease: an NIH workshop report. J Clin Invest 2023; 133: e171582. 37655664 10.1172/JCI171582 PMC10471172

[22] Koedam TWA, Bootsma BT, Deijen CL, et al. Oncological outcomes after anastomotic leakage after surgery for colon or rectal cancer: increased risk of local recurrence. Ann Surg 2022; 275: e420–7. 32224742 10.1097/SLA.0000000000003889

